# Characterization and putative post-translational regulation of α- and β-tubulin gene families in *Salix arbutifolia*

**DOI:** 10.1038/srep19258

**Published:** 2016-01-12

**Authors:** Guodong Rao, Yanfei Zeng, Caiyun He, Jianguo Zhang

**Affiliations:** 1 State Key Laboratory of Tree Genetics and Breeding, Research Institute of Forestry, Chinese Academy of Forestry, Beijing 100091, Republic of China; 2 Collaborative Innovation Center of Sustainable Forestry in Southern China, Nanjing Forestry University, Nanjing 210037, Republic of China; 3 Key Laboratory of Tree Breeding and Cultivation, State Forestry Administration, Research Institute of Forestry, Chinese Academy of Forestry, Beijing 100091, Republic of China

## Abstract

Microtubules, which are composed of heterodimers of α-tubulin (TUA) and β-tubulin (TUB) proteins, are closely associated with cellulose microfibril deposition and play pivotal roles in plant secondary cell wall development. In the present study, we identified eight *TUA* and twenty *TUB* genes in willow (*Salix arbutifolia*). Quantitative real-time PCR analysis showed that the small number of *TUA* gene family members relative to that of *TUBs* was complemented by a higher transcript copy number for each *TUA* gene, which is essential to the maintenance of the tubulin 1:1 heterodimer assembly. In *Salix*, five of eight TUAs were determined to be unusual because these contained a C-terminal methionine acid, leucine acid, glutamic acid, and glutamine acid, instead of the more typical tyrosine residue, which in turn generated the hypothesis of post-translational modifications (PTMs) that included deleucylation, demethiolation, deglutamynation, and deaspartylation. These PTMs are responsible for the removal of additional amino acid residues from TUAs prior to detyrosination, which is the first step of C-terminal PTMs. The additional PTMs of the *TUA* gene family might be responsible for the formation of different tubulin heterodimers that may have diverse functions for the adaptation of the woody perennial growth for *Salix*.

Cortical microtubules (MTs), which consist of heterodimers of α-tubulin (TUA) and β-tubulin (TUB) proteins, are essential to the plant cell morphogenesis and play a key role in guiding the deposition of cellulose microfibrils during plant cell wall formation^1^,^2^. The amino acid sequences of the TUA and TUB subunits show about 88% homology in animals^3^,^4^,^5^, plants^6^, protists^7^,^8^ and fungi^9^. The *TUA* and *TUB* genes in plants have been extensively investigated; for example, *Arabidopsis thaliana* harbors six *TUA* genes that encode four distinct proteins^10^,^11^ and at least nine *TUB* genes that encode nine proteins^12^. On the other hand, cotton (*Gossypium hirsutum*) contains five *TUA* genes^13^ and nineteen *TUB* genes^14^; rice (*Oryza sativa*) has four *TUA* genes^15^,^16^ and eight *TUB* genes^17^, and *Populus trchocarpa* possesses eight *TUA* and twenty *TUB* genes^18^.

The expression pattern of plant tubulin has also been studied in various species. The *Arabidopsis AtTUA1* gene is only expressed in flowers, and *AtTUA2*–*AtTUA5* are transcribed in leaves, roots, and flowers^10^,^19^. Of the nine of *AtTUB* genes, the transcripts of *AtTUB5* and *AtTUB6* preferentially accumulate in leaves and petioles^12^, whereas the *AtTUB1* transcript is primarily expressed in the roots^20^, and the other seven *TUBs* are expressed in vegetative tissues. Rice *OsTUB8* is predominantly expressed in flowers and other seven *OsTUBs* are differentially expressed during development^17^. The transcript levels of five cotton *TUAs* are much higher in fibers than that in various other tissues, including pollen^13^, and 9 of 19 *GhTUB* genes are preferentially expressed in cotton fiber cells^14^. *Populus PtTUA1*, *PtTUA5*, *PtTUA7*, *PtTUB5/6*, *PtTUB7*, *PtTUB8*, *PtTUB19*, and *PtTUB20* have the highest transcript levels in pollen, whereas the other *PtTUAs* and *PtTUBs* are upregulated in the xylem^18^. Functionally distinct microtubule subtypes are generated in cells through the expression of different tubulin isotypes and through post-translational modifications (PTMs). In animals, tubulins have different homologs that undergo various PTMs such as tyrosination/detyrosination, acetylation, polyglutamylation, and polyglycylation, which in turn lead to the appearance of various tubulin isoforms and classes of MTs^21^,^22^,^23^,^24^,^25^,^26^,^27^. In plants, a large number of tubulin isotypes have been isolated from different species, whereas investigations on PTMs in plant tubulin are limited.

Willow (*S. arbutifolia*) is a fast-growing tree and is cold-resistant, thus rendering it an ideal material for biomass production research. The interest of using willow for biomass production is growing, thereby resulting in increased pressure on breeding high yielding and resistant clones that are adapted to different environments^28^. MTs play central roles in several basic processes of eukaryotic cells, which include cell division, cell motility, intracellular transport, and cell elongation. The long-term goal of our investigations is to characterize the mechanisms involved in the genetic control of MT function in woody plants. In the present study, we report the cloning, sequencing, and analysis of gene structure, phylogenetic diversity, and expression patterns of eight *TUA* genes and twenty *TUB* genes in *S. arbutifolia*. We also analyzed the amino acid residues at the C-terminal region of the TUA and TUB proteins, and our results have prompted us to speculate that the other PTMs are related to the new C-terminal amino acid residues in *Salix.*

## Materials and Methods

### Identification of α- and β-tubulin genes in *Salix*

The whole-genome sequence data of *Salix. suchowensis* were used for the identification of α- and β-tubulin genes, including both of DNA and CDS, via reciprocal BLAST analysis using protein sequence of 20 *Populus* and 15 *A. thaliana* tubulin genes^29^. *Salix* homologs to the 20 *Populus* tubulin genes were identified by using BLASTP, with the e-value cut-off set at 1-E03. The same protocol was performed for the detection of willow homologs by using the 15 *A. thaliana* tubulin genes.

### DNA cloning and sequencing

Total RNA was extracted and treated with RNase-free Dnase (Promega, Madison, USA) to remove contaminating DNA. Purification of first-strand cDNA was conducted following the protocol of Lu *et al.*^30^. Primers (Supplementary file 1: Table S1) were designed according to the sequences of the α- and β-tubulin genes, which were obtained by analyzing the *Salix* genome. PCR was performed as follows: 94 °C for 2 min, followed by 30 cycles of 94 °C for 30 s, 56 °C for 45 s, and 72 °C for 2 min. The PCR products were cloned into the pMD18-T vector (Takara, Japan, http://www.takara.com.cn) and sequenced. The 28 cDNAs (8 TUAs and 20 TUBs) from *Salix* were designated as SaTUA1–SaTUA8 and SaTUB1–SaTUB20, respectively.

### Real-time PCR Analysis

Stem developing phloem, full expanded leaves, stem developing xylem, shoot tips (1.0 cm–1.5 cm from the top of the plant), and inflorescence were obtained from three 1-year-old *S. arbutifolia*, which were growing at the Baishilazi National Nature Reserve of China. Real-time PCRs were conducted using the SYBR Green Perfect (Takara, Dalian, China) and StepOnePlus^TM^ System (Applied Biosystems). All of the PCR products were sequenced and the dissociation curve was analyzed to verify amplification specificity^31^. The purified PCR products were employed to generate a standard curve to establish the quantitative correlation between the CT values and the transcript copy numbers^31^. Each qRT-PCR reaction was repeated at least three times, and each standard curve comprised at least 5 points. The transcript levels of the samples did not significantly vary (*P*-value ≥0.95) from the calculated average of the biological replicates.

### Sequence analysis

Phylogenetic analysis was performed using the software, MEGA 5^32^. The phylogenetic relationships of the gene models were evaluated using the neighbor-joining or minimum-evolution tree with 1,000 bootstrap trials. The full-length amino acid sequences were aligned by using ClustalW^33^ and displayed with DNAMAN. The exon-intron structures were displayed by using the Gene Structure Display Server (GSDS, http://gsds.cbi.pku.edu.cn/index.php).

## Results

### *In silico* identification of *Salix TUA* and *TUB* genes

The present study identified a total of eight *TUA* genes, which were designated *SaTUA1* through *SaTUA8*, and 20 *TUB* genes, namely, *SaTUB1* to *SaTUB20*. The size of the predicted open reading frames of the eight *TUA* genes ranged from 1,350 bp to 1,356 bp, whereas that of the *TUB* genes ranged from 1,335 to 1,356 bp. The eight *TUA* cDNAs encode eight distinct TUA proteins, whereas the 20 *TUB* cDNAs encoded 19 TUB proteins, mainly because *TUB7* and *TUB12* encoded the same protein. The length of the TUA proteins ranged from 449 to 451 amino acids, whereas that of the TUB proteins ranged from 444 to 451 amino acids. The *Salix TUAs* shared 73.9% to 94.5% cDNA sequence and 88.6% to 98.4% protein sequence identity (Supplementary file 2: Figure S1, Table S2), whereas the *TUBs* shared 74.6% to 99.8% cDNA and 86.8% to 99.1% (except for SaTUB7/12) protein sequence identity (Supplementary file 3: Figure S2, Table S3).

**Figure 1:**
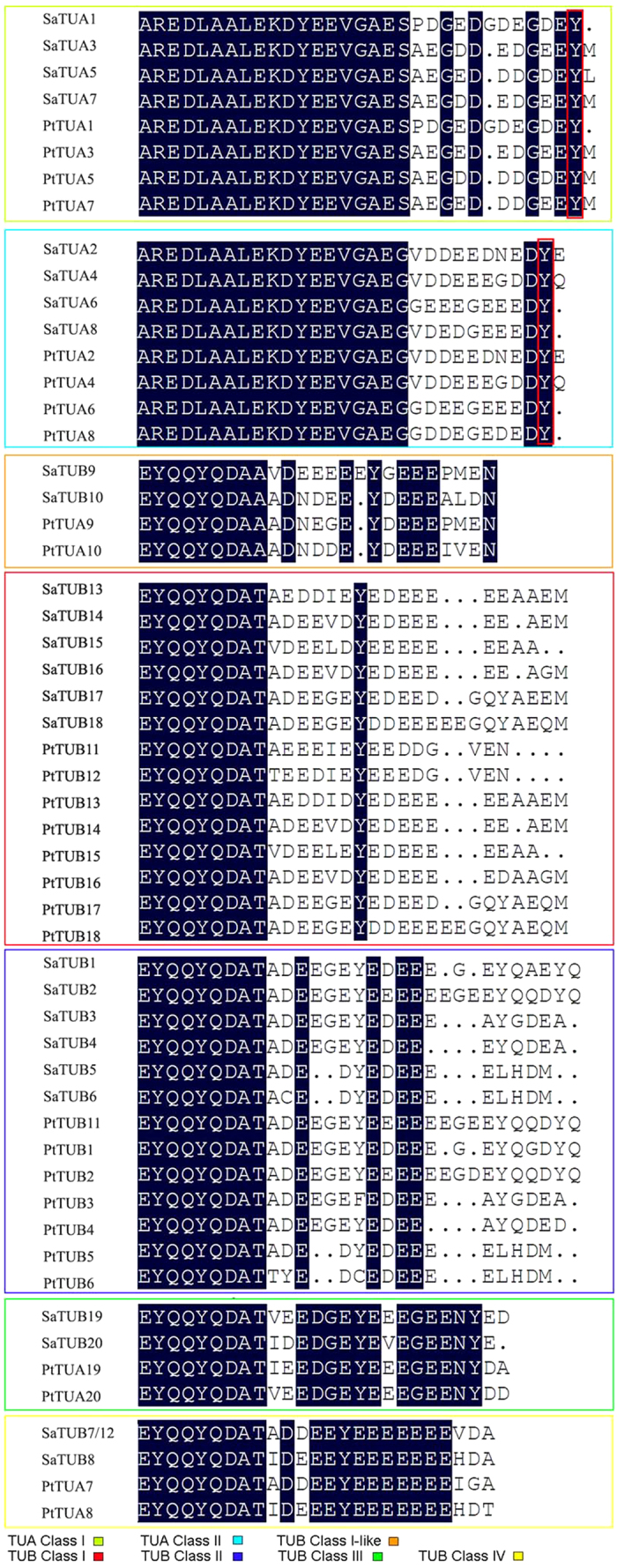
Alignment of TUA and TUB C-terminal amino acid sequences of *Salix and Populus*. Different colored boxes indicate various classes of TUAs. The GenBank accession numbers or locus of JGI of the *Populus* is provided in Supplementary file 4: Table S4.

Three functional domains in TUA and TUB were characterized using electron crystallography: the N-terminal domain, which contained the GTP binding site; the C-terminal domain, which comprised microtubule-associated proteins (MAPs); and the motor protein binding region and an intermediate domain containing the Taxol binding site^34^. PTMs are essential for the maturation of the tubulin protein, which include modifications such as tyrosination/ detyrosination^35^, acetylation^36^, polyglutamylation^37^, phosphorylation, and polyglycylation^38^. Except for acetylation, all modifications take place in the hypervariable C-terminal region^39^. The *Salix* C-terminal region consisted of about 20 amino acid residues that constitute a major variable domain for TUB, and to a lesser extent, for TUA as well. Specific differences in the C-terminal domain were detected among *Salix* and other plants, and most plant TUA proteins are of the Y-type, wherein the last amino acid is a Tyr residue and is involved in PTMs of tyrosination/detyrosination. Three of the eight *Salix* TUA proteins are of the Y-type, whereas the other five terminates are TUA3-Met and TUA7-Met (M-type), TUA5-Leu (L-type), and TUA2-Glu and TUA4-Gln (E/Q-type). A different residue between *Salix* and *Populus* was observed, and the last amino acid of TUA5 in *Salix* was a Leu residue, whereas in *Populus*, this was a Met residue (Fig. 1).

### Cloning of willow *TUA* and *TUB* genes

**Figure 2:**
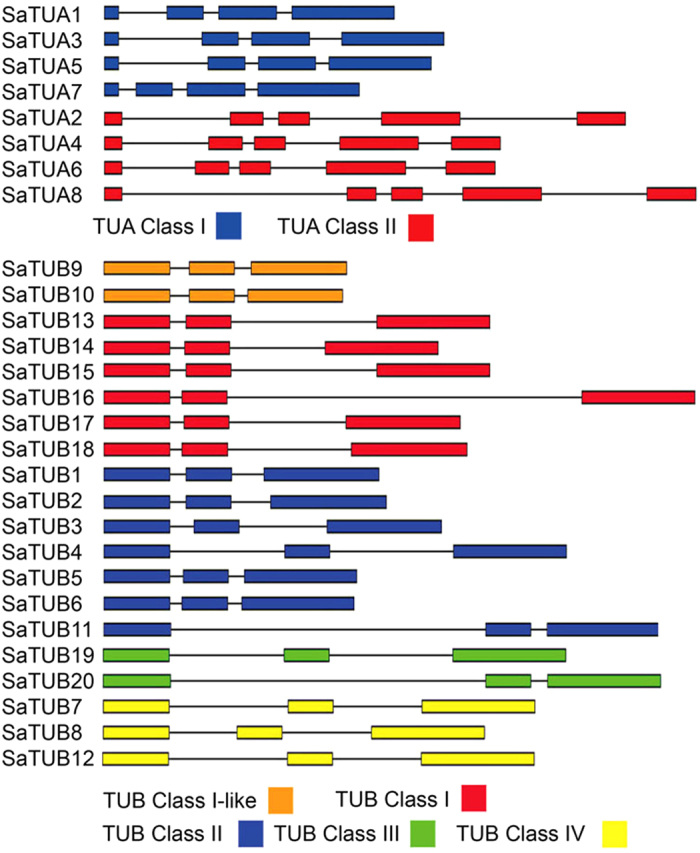
*Salix TUA* and *TUB* gene structures. Different colors indicate various classes. **(A)** Classes I and II of *TUAs*. **(B)** Class I, class I-like, class II, class III, and class IV of *TUBs*.

*TUA* and *TUB* genes were isolated from three different willow species, namely, *S. arbutifolia*, *S. matsudana*, and *S*. *matsudana* var. *tortuosa* (corkscrew willow). No sequence differences were observed among these species (GenBank accession numbers of *Salix TUAs* are from KC238439 to KC238446, and the accession number of *TUBs* are from KC243679 to KC243698). The eight *TUA* genes were divided into two classes, namely, class I and class II, which differed both in the number and position of the exons. Class I *TUA* genes comprised four exons, whereas those of class II consisted of five exons (Fig. 2). In each class, the sizes of the exons were conserved compared to their homologs in the same position. *Populus TUA* genes (*PtTUA1–PtTUA8*) and *Salix TUA* genes (*SaTUA1–SaTUA8*) have the same number and position of exons, whereas these differ in terms of the size of introns, thereby resulting in variations in gene size. Compared to *TUA* class I genes, *TUA* class II genes formed long introns, particularly *TUA2* and *TUA8,* which result in large-sized of *TUA2* and *TUA8* genes. *SaTUB* genes are divided into five classes; all of these have three exons, and the size of each exon is the same, whereas two introns are located at a conserved position, which is analogous to the gene structure of *Arabidopsis* and *Populus TUB* gene family^10^,^18^. Class I *TUB* genes were bigger in size compared to class I-like *TUB* genes because the second intron in class I were longer than that in class I-like. *SaTUB11* is much longer than other *SaTUBs* in class II because of the longer first intron. *SaTUB19* and *SaTUB20* genes were longer than their counterpart *PtTUB19* and *PtTUB20* genes in class III, and *SaTUB7* gene was longer than *SaTUB8*, which shows the opposite features compared to *Populus* in class IV.

### Phylogenetic analysis of the TUA and TUB families

**Figure 3:**
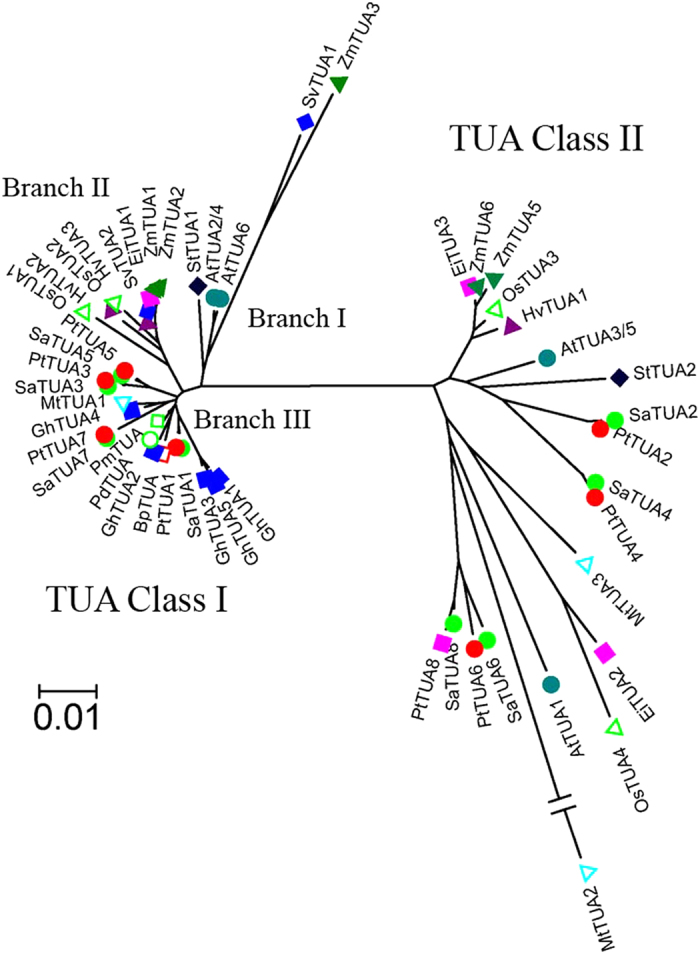
Phylogenetic analyses of plant TUA proteins. Unrooted phylogenetic tree of TUA from *S. arbutifolia* (Sa), *Populus trchocarpa* (Pt), *Arabidopsis thaliana* (At), *Betula pendula* (Bp), *Medicago truncatula* (Mt), *Eleusine indica* (Ei), *Gossypium hirsutum* (Gh), *Hordeum vulgare* (Hv), *Oryza sativa* (Os), *Prunus dulcis*(Pd), *Pseudotsuga menziesii* (Pm), *Setaria viridis* (Sv), *Solanum tuberosum* (St), *and Zea mays* (Zm). GenBank accession numbers or locus (*Populus*) of the sequences are provided in Supplementary file 5: Table S6. Different colors indicate various species.

The results of hylogenetic analysis of plant TUA proteins are presented in Fig. 3. Plant TUAs were divided into two classes, Class I and Class II, with the eight *Salix*, four rice, eight *Populus*, and six *Arabidopsis* isoforms equally distributed between the two classes. Each *Salix* TUA has a corresponding homologue in *Populus*, and the homologs share 89.3%–99.7% amino acid sequence identity (Supplementary file 4: Table S5), indicating that each pair of *Salix* and *Populus* TUAs originated from the same ancestor. In class I, most monocot and dicot TUAs were separately clustered. In the second branch of TUA class I, all of the TUAs are monocots, which include two *Zea mays* TUAs (ZmTUA1 and ZmTUA2), two *Oryza sativa* TUAs (OsTUA1 and OsTUA2), a *Setaria viridis* TUA (SvTUA2), a *Eleusine indica* TUA (EiTUA1) and two *Hordeum vulgare* TUAs (HvTUA2 and HvTUA3). In the third branch of TUA class I, all of the TUAs are dicots, which include eight TUAs from *Salix* and *Populus*, five TUAs from *Gossypium hirsutum* and four TUAs from *Betula pendula*, *Pseudotsuga menziesii*, *Medicago truncatula*, and *Prunus dulcis*. The first branch of TUA class I is a mixed branch, which included both monocot and dicot species, indicating that the *TUA* genes in this branch were more original than the second and the third branch *TUA* genes. The xylem-originating SaTUA1 was clustered with PtTUA1, GhTUA2, GhTUA3, and GhTUA4 from cotton, which is involved in secondary cell wall development^13^. In class II, most of the monocot and dicot TUAs were also separately clustered into different branches such as the five monocot TUAs that were clustered in the first branch, and dicot SaTUA2/4, which were more closely clustered with dicot AtTUA3/5, PtTUA2/4, and StTUA2 in the second branch.

**Figure 4:**
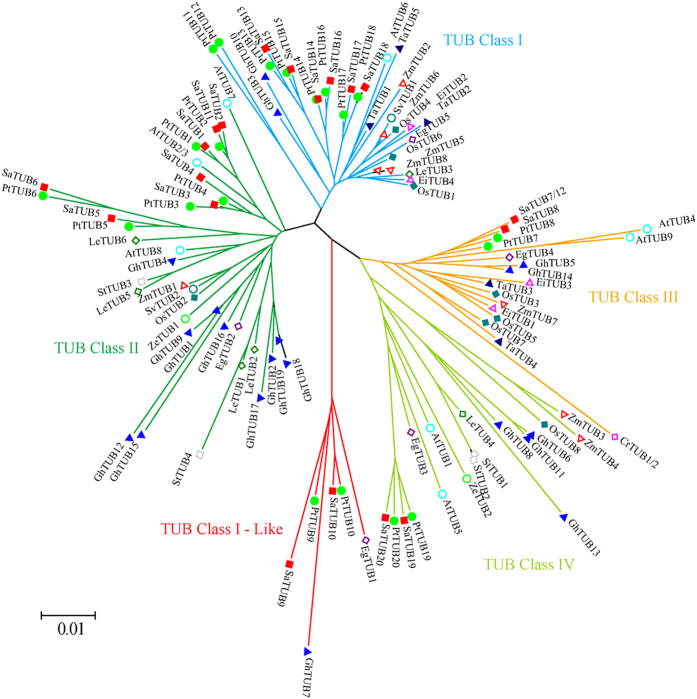
Phylogenetic analysis of plant TUB proteins. Unrooted phylogenetic tree of TUB from *S. arbutifolia* (Sa), *Zinnia elegans* (Ze), *Triticum aestivum* (Ta), *Populus trchocarpa* (Pt), *Arabidopsis thaliana* (At), *Betula pendula* (Bp), *Eucalyptus grandis* (Eg), *Lycopersicon esculentum* (Le), *Eleusine indica* (Ei), *Gossypium hirsutum* (Gh), *Hordeum vulgare* (Hv), *Oryza sativa* (Os), *Prunus dulcis*(Pd), *Pseudotsuga menziesii* (Pm), *Setaria viridis* (Sv), *Solanum tuberosum* (St), and *Zea mays* (Zm). The five classes are indicated in different colors; the GenBank accession numbers of the sequences are provided in Supplementary file 5 Table S6.

The SaTUBs and PtTUBs shared 88.4% to 100% amino acid sequence identity (Supplementary file 4: Table S5), and have similar properties of number and position of exons (Fig. 2), suggesting that the *Salix* TUBs and *Populus* TUBs were derived from the same ancestor. However, not every *Salix* TUB has a homolog in *Populus*, which suggests that *Salix* and *Populus* TUBs separately underwent a different type of expansion. The phylogenetic NJ tree is presented in Fig. 4 shows the classification of plant TUBs into five groups, namely, Class I, Class I-like, Class II, Class III, and Class IV. Five *Salix* TUBs showed no homologs in *Populus*, which included TUB3, TUB4, TUB7/12, TUB8, and TUB11, and were clustered in classes II and IV. Class I and Class I-like comprise the largest woody plant cluster that mainly consist of *Salix* (8 out of 19) and *Populus* (10 out 20) TUB family members. PtTUB11 paired with PtTUB12 in the class I-like group, whereas SaTUB11 showed no homolog in *Salix*, which further indicates that PtTUB12 was derived from PtTUB11 via tandem/terminal fusion or translocations. On the other hand, SaTUB11 underwent a different evolutionary route compared to PtTUB11 and PtTUB12 after the salicoid genome-wide gene duplication. In class I-like, EgTUB1 was possibly involved in the determination of the orientation of cellulose microfibrils in plant secondary fiber cell walls^40^, and the inclusion of *Salix* TUB9 and TUB10 in this group suggests that these two TUBs might have evolved for secondary cell wall development. Classes III and IV each contain two *Salix* TUBs, together with two *Populus* and two *Arabidopsis* isoforms that were derived from genome-wide duplications.

### Gene-specific transcript abundance of the TUA and TUB family in *Salix*

**Figure 5:**
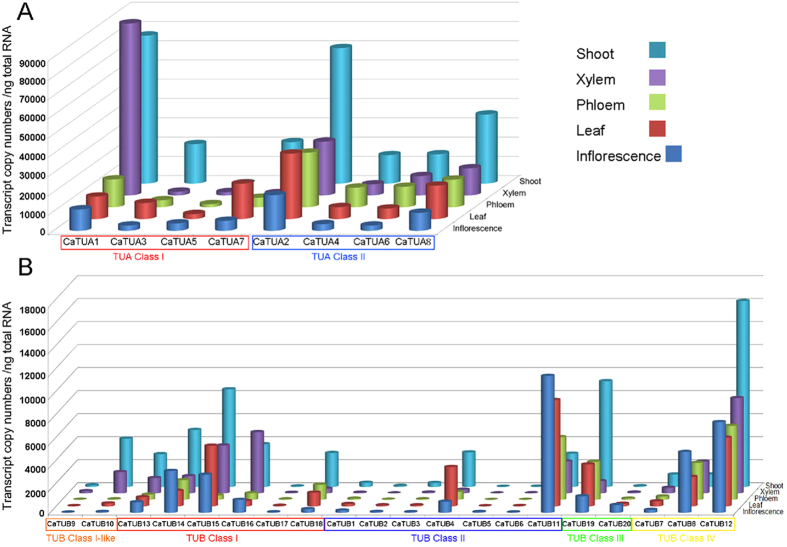
qRT-based tissue-specific transcript levels in various *Salix* tissues. Tissues were collected from three trees, and three biological replicates were prepared from each sample. Mean transcript copy numbers and standard error of the means from three technical replicates are small. (**A)** Transcript copy numbers of *TUA* genes in each nanogram of total RNA from different tissues. (**B)** Transcript copy numbers of *TUB* genes in each nanogram of total RNA from different tissues.

The gene-specific qRT-PCR primers were designed based on the 3’-UTRs of the eight TUA and twenty TUB genes, and stem developing phloem, fully expanded leaf, stem developing xylem, shoot tip, and inflorescence were analyzed by quantitative real-time PCR. Transcripts of all *TUA* members, with the exception of *TUA5*, were abundant in shoot. The class I TUA members are comparatively less abundant in all of the tissues compared to the class II *TUA* members. In class I, *TUA1* showed the most abundant transcript in the developing xylem and was also expressed at moderate levels in the phloem, leaves, and inflorescences. The M-type *TUA3* and *TUA7* were shoot- and leaf-specific, whereas the L-type *TUA5* was less abundant in all tissues (Fig. 5). In class II, *TUA2* was highly expressed in all of the tissues, followed by *TUA8*, whereas *TUA4* and *TUA6* were downregulated. In general, the Y-types *TUA1*, *TUA6*, and *TUA8* were abundant in the xylem, suggesting that these originated as a xylem-specific cDNAs. The transcript pattern of *Salix TUBs* differed from that of *TUAs*. TUB class I, which included *TUB13*–*18*, and TUB class IV, which consisted of *TUB7*, *TUB8*, and *TUB12*, showed high transcript levels compared to that of the other *TUB* genes. *TUB12*, *TUB14*, *TUB15*, and *TUB19* represented the predominant *TUB* species in shoot tips, followed by *TUB10* and *TUB13*, and *TUB8* and *TUB11* were most abundant in inflorescences. The expression pattern of each pair of *TUB* paralogs differed such as the pair *TUB9* and *TUB10*, wherein *TUB9* was not abundant in all tissues, whereas *TUB10* was upregulated in the xylem and shoots. In the case of paralog pair *TUB19* and *TUB20*, *TUB19* was upregulated in all tissues compared to *TUB20*, which was only abundant in inflorescences. Such differences in expression pattern between *Salix* paralogs were suggestive of the functional divergence or redundancy of *TUB* genes.

## Discussion

Six *TUA* and nine *TUB* genes from *Arabidopsis* and five *TUA* and nineteen *TUB* genes from cotton expanded into eight *TUA* and twenty *TUB* genes in *Populus* and *Salix,* and together with the phylogenetic analysis of their deduced amino acid sequence, suggest that the *Salix TUA* and *TUB* genes underwent different expansions via eurosid genome-wide gene duplication and salicoid genome-wide gene duplication events, followed by reciprocal tandem/terminal fusion, translocations, and other chromosomal rearrangements. The *Salix* and *Populus TUA* and *TUB* genes have identical features, including gene numbers, gene structure, and amino acid changes at the C terminus, together with the very high levels of sequence identity, which demonstrate that the Salicaceae TUAs and TUBs were derived from the salicoid genome-wide gene duplication event, which was estimated to have occurred sometime between 6 and 10 million years ago^41^.

Plants are unable to tolerate large imbalances in the ratio of TUA to TUB within the cytoplasm. In maize, when a single tubulin gene was transformed and overexpressed, the plant did not regenerate, but when both *TUA* and *TUB* were transformed and the two heterodimer subunits were overexpressed, regeneration occurred^42^,^43^. In aspen, the transgenically manipulated *TUA1* expression also failed to produce viable transformants^18^. The lethality caused by subunit imbalance seems to be a general phenomenon. In mammals and in yeast, overexpression of *TUA* or *TUB* leads to cell-cycle arrest and reduced cell viability^44^,^45^. In sum, the balance in the expression level of both TUA and TUB are essential to the tubulin 1:1 heterodimer assembly, which is crucial for the growth and development of plants and animals. In mammals, the TUA and TUB families are identical in size, seven genes of each family were identified in human and mouse^46^,^47^, thus facilitating the formation of the tubulin 1:1 heterodimer. On the other hand, in the present study involving *Salix*, the size of the of the TUA and TUB families differed (eight TUAs and nineteen TUBs), thus rendering a more difficult situation for the formation of the 1:1 heterodimer, because of the higher number of TUB genes compared to that of the TUAs. The mechanism responsible for the difference in size of the two families in *Salix* is unknown; however, there appears to be a solution to the establishment of the 1:1 heterodimer assembly. The transcript copy numbers between *TUAs* and *TUBs* are markedly different, wherein the transcript copy number of *TUAs* ranged from 153 to 89,271 per nanogram of total RNA, whereas that of the *TUBs* ranged from 4 to 16,156 per nanogram of total RNA. This observation indicates that the *TUAs* have multiple-fold higher number of transcripts than *TUBs* (Fig. 5), which apparently compensates for the lower number of members in the *TUA* gene family.

**Figure 6:**
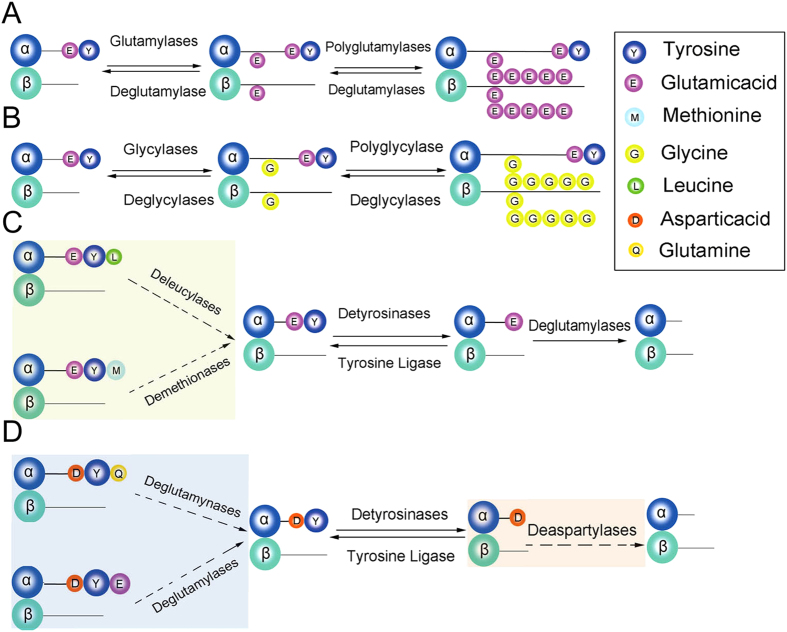
Putative tubulin C-terminal PTMs in *Salix*. Schematic representation of the generation and removal of each tubulin PTMs with corresponding enzymes, and amino acids involved in PTMs are shown in the box at the top right corner. **(A)** Glutamylation and polyglutamylation. **(B)** Glycylation and polyglycylation. **(C)** Putative deleucylation and demethiolation. **(D)** Putative deglutamynation, deglutamylation and deaspartylation.

Cells generate distinct microtubule subtypes by two ways, namely, by expression of different tubulin isotypes and by PTMs such as detyrosination and further cleavage to Δ2-tubulin, acetylation, polyglutamylation, and polyglycylation^48^. Except for acetylation, all of the tubulin PTMs occurs at the C terminal tails, which are exposed at the outer surface of microtubules of TUA and TUB (Fig. 6). Molecular cloning of tubulin genes showed that a C-terminal Tyr is encoded by most *TUA* genes^49^, the tyrosination–detyrosination cycle is initiated by the removal of a Tyr functional group (detyrosination), and re-addition of Tyr (tyrosination) serves to reverse the modification and to return tubulin to its initial state^49^,^50^. *Salix* TUAs have a distinct C-terminal residue compared to the other species, and four different types of TUAs were identified in the present study, namely, three Y-types, which include M-type, L-type, and E-type, and the Q-type. Except for the three Y-type TUAs, the second to the last amino acid of the other TUAs are still the Y residues (Fig. 1).

Detyrosination is the first step of C-terminal PTM, and it increases the stability and longevity of MTs. It is also essential for motors and non-motor MAPs binding to MTs^51^,^52^. In *Salix*, the additional amino acids are blocks of detyrosinases, and the tubulin heterodimer requires other enzymes to remove these amino acid residues to achieve the detyrosinate state. We hypothesized that in *Salix*, other PTMs are responsible for the removal of the additional amino acid residues prior to detyrosination; therefore, the none Y-type TUAs were detyrosinated after completion of the other putative PTMs. These PTMs are termed deleucylation, demethiolation, deglutamynation, and deglutamylation (Fig. 6). The putative deleucylases possibly removes the Leu residue present at the C-terminal of *TUA5*, demethiolation removes Met residues at the C-terminal of *TUA3*, *TUA7*, and the putative deglutamynases apparently catalyzes the removal of Gln residues of the C-terminal of *TUA4*. Detyrosinated tubulin can be further converted to Δ2-tubulin by removal of a Glu residue from the C-terminal, thereby exposing this region after detyrosination that is catalyzed by deglutamylase enzymes^25^,^53^,^54^. Δ2-tubulin generally occurs at the final stages of PTMs, and Δ2-modification irreversibly locks microtubules in a detyrosinated state, thereby excluding the MTs from depolymerization-polymerization cycles^55^,^56^. Similarly, Δ2-modification has the biochemical function of regulating MT dynamics and stability. In the *Salix* TUA family, two distinct amino acid residues occur right before the Y residue; in class I, the residue is Glu, whereas in class, this corresponded to the Asp residue. The Glu residue can be removed by deglutamylase enzymes, thereby forming Δ2-tubulin^25^, whereas no enzyme catalyzed Asp to form Δ2-tubulin, and the Asp showed similar chemical properties to that of Glu, wherein both are acidic and electronegative. The putative deaspartylation involves the removal of the Asp residue, thereby forming Δ2-tubulin, which is similar to that of deglutamylation as presented in Fig. 6. These additional PTMs of the *TUA* gene family facilitate in the formation of different tubulin heterodimers that provides functional diversity for adaptation for the woody perennial growth of *Salix* in its natural habitat.

## Additional Information

**How to cite this article**: Rao, G. *et al.* Characterization and putative post-translational regulation of α- and β-tubulin gene families in *Salix arbutifolia*. *Sci. Rep.* **6**, 19258; doi: 10.1038/srep19258 (2016).

## Supplementary Material

Supplementary Dataset 1

Supplementary Dataset 2

Supplementary Dataset 3

Supplementary Dataset 4

Supplementary Dataset 5
